# Association Between Neonatal Neuroimaging and Clinical Outcomes in Zika-Exposed Infants From Rio de Janeiro, Brazil

**DOI:** 10.1001/jamanetworkopen.2019.8124

**Published:** 2019-07-31

**Authors:** Kara-Lee Pool, Kristina Adachi, Stellios Karnezis, Noriko Salamon, Tahmineh Romero, Karin Nielsen-Saines, Sheila Pone, Marcia Boechat, Mitsue Aibe, Tallita Gomes da Silva, Carla Trevisan Martins Ribeiro, M. Ines Boechat, Patricia Brasil, Andrea Zin, Irena Tsui, Stephanie L. Gaw, Pedro Daltro, Bianca Guedes Ribeiro, Tatiana Fazecas, L. Celso Hygino da Cruz, Renata Nogueira, Zilton Vasconcelos, Jose Paulo Pereira, Tania Saad Salles, Claudia Neves Barbosa, Weiqiang Chen, Suan-Sin Foo, Jae Jung, Maria Elisabeth Moreira, Marcos Pone

**Affiliations:** 1 David Geffen School of Medicine, University of California, Los Angeles; 2 Fundação Oswaldo Cruz, Rio de Janeiro, Brazil; 3 University of California San Francisco School of Medicine; 4 Clinica de Diagnostico por Imagem CDPI, Rio de Janeiro, Brazil; 5 University of Southern California School of Medicine, Los Angeles

## Abstract

**Importance:**

Congenital Zika virus (ZIKV) infection may present with a spectrum of clinical and neuroradiographic findings.

**Objective:**

To determine whether neuroimaging findings for infants with a history of ZIKV exposure are associated with infant clinical outcomes and gestational age at antenatal ZIKV infection.

**Design, Setting, and Participants:**

This cohort study retrospectively reviewed neuroimaging results (computed tomography and/or magnetic resonance imaging scans) of 110 ZIKV-exposed infants from a maternity and children’s hospital in Rio de Janeiro, Brazil, following the 2015 to 2016 ZIKV epidemic. Neuroimaging from March 1, 2016, to June 30, 2017, was evaluated to determine whether findings were associated with clinical outcomes and the timing of maternal ZIKV infection. Data were analyzed from July 1, 2017, to August 30, 2018.

**Exposures:**

Neuroimaging (computed tomography and/or magnetic resonance imaging) was performed on ZIKV-exposed infants after birth. Blood and/or urine specimens from mothers and infants were tested for ZIKV by polymerase chain reaction assay.

**Main Outcomes and Measures:**

Neuroimaging studies were evaluated for structural abnormalities and other forms of brain injury.

**Results:**

A total of 110 infants with a mean (SD) gestational age of 38.4 (2.1) weeks had neuroimaging and clinical outcome data reviewed. Of these, 71 (65%) had abnormal neuroimaging findings, with the majority (96%) classified as having severe ZIKV infection at birth. The most common neuroimaging abnormalities were structural abnormalities including brain calcifications, especially at the cortico-subcortical white matter junction, cortex malformations, ventriculomegaly, and reduced brain volumes, followed by brainstem hypoplasia, cerebellar hypoplasia, and corpus callosum abnormalities. Frequency of abnormal imaging was higher in infants with specific clinical findings as opposed to those without them; these findings included fetal brain disruption sequence (100% vs 35%), microcephaly (100% vs 30%), congenital contractures (100% vs 58%), ophthalmologic abnormalities (95% vs 44%), hearing abnormalities (100% vs 58%), and neurologic symptoms (94% vs 10%). Four of 39 infants (10%) without initial evidence of severe ZIKV infection and normal findings on neurologic evaluation at birth had abnormal neuroimaging findings. Neuroimaging abnormalities differed by trimester of maternal ZIKV infection, with 63% of infants born to mothers infected in the first trimester, 13% of infants born to mothers infected in the second trimester, and 1% of infants born to mothers infected in the third trimester exhibiting neuroimaging abnormalities. The odds of abnormal neuroimaging were 7.9 times greater for infants with first trimester ZIKV exposure compared with other trimesters combined (odds ratio, 7.9; 95% CI, 3.0-20.4; *P* < .001).

**Conclusions and Relevance:**

Neuroimaging abnormalities of computed tomography and/or magnetic resonance imaging scans were common in ZIKV-exposed infants. While neuroimaging abnormalities were seen in 10% of infants without clinically severe ZIKV, most occurred almost exclusively among those with clinically severe ZIKV, especially among those with a history of ZIKV exposure in the first trimester.

## Key Points

**Question:**

Are neuroimaging findings of infants exposed to Zika virus associated with infant clinical outcomes and gestational age of antenatal Zika virus infection?

**Findings:**

In this cohort study of 110 infants with confirmed or suspected antenatal exposure to Zika virus evaluated at a referral center from 2015 to 2016, 96% of abnormal neuroimaging occurred among Zika virus–exposed infants with severe clinical findings at birth; however, 10% of infants without severe clinical manifestations also had neuroimaging abnormalities. In addition, an increased risk of abnormal imaging was associated with Zika virus exposure in the first trimester compared with later trimesters.

**Meaning:**

Neuroimaging of infants exposed to Zika virus is an important part of evaluating infants with a history of Zika virus in utero exposure, particularly for those exposed in the first trimester.

## Teaser

This cohort study examines the association between neonatal neuroimaging findings and clinical outcomes in infants exposed to Zika virus in utero in Brazil.

## Introduction

Zika virus (ZIKV) infection during pregnancy can cause a spectrum of central nervous system (CNS) abnormalities.^1,2,3^ Infants severely affected by ZIKV classically present with microcephaly, other structural brain abnormalities, ocular abnormalities, congenital contractures, and profound neurologic impairment.^2^ Clinical findings of ZIKV-exposed neonates with severe congenital ZIKV have been well described, including among several neuroimaging reports,^2,3,4,5,6^ but the utility of neuroimaging for less severely affected infants is unclear. This study describes neuroimaging (computed tomography [CT] and/or magnetic resonance imaging [MRI]) findings and clinical outcomes in ZIKV-exposed infants with a range of clinical findings at birth. Our primary aims were as follows: (1) to review neuroimaging of infants with antenatal ZIKV exposure and to determine whether neuroimaging (CT and/or MRI) findings were associated with infant clinical outcomes and (2) to determine whether the gestational age at in utero ZIKV infection was associated with neuroimaging findings.

## Methods

### Study Population

A retrospective review of available neuroimaging (CT and/or MRI) was performed and correlated with clinical data obtained from March 1, 2016, to June 30, 2017, for ZIKV-exposed infants followed at the Fernandes Figueira Institute (IFF), Oswaldo Cruz Foundation in Rio de Janeiro, Brazil. The IFF is a high-risk obstetric and pediatric referral center. Infants with laboratory-confirmed ZIKV exposure during pregnancy or presumed congenital ZIKV infection (based on clinical history and evaluation) with follow-up outcome data available were included. Data are reported according to the Strengthening the Reporting of Observational Studies in Epidemiology (STROBE) reporting guideline for cohort studies. Institutional review board approvals were obtained at both the Oswaldo Cruz Foundation and the University of California, Los Angeles for retrospective review of patient medical records with a waiver of informed consent. Mothers and infants followed up at IFF for whom blood was drawn for ZIKV laboratory diagnostics provided written informed consent.

### Laboratory Testing

Laboratory confirmation of ZIKV infection was performed using real-time reverse transcriptase–polymerase chain reaction (RT-PCR) assays with the ZIKV QuantiTect Probe RT-PCR Kit (Qiagen) at the Oswaldo Cruz Foundation IFF laboratory. Testing was performed on mothers during pregnancy and infants at birth from serum and/or urine. Timing of maternal infection during pregnancy was defined as the gestational week in which women had a positive ZIKV PCR result in blood and/or urine. In the absence of a positive maternal PCR result, it was the time during pregnancy in which women had clinical findings of ZIKV infection.

### Infant Clinical Assessments

During the first year of life, infants were evaluated by pediatric infectious disease specialists at birth and monthly and by pediatric neurologists and geneticists at birth. All infants had serologic and/or molecular testing for other prenatal infections including dengue, chikungunya, parvovirus B19, cytomegalovirus, toxoplasmosis, rubella, HIV, and syphilis. Preterm birth was defined as gestational age of less than 37 weeks. Neuroimaging was performed on infants after birth to evaluate for CNS abnormalities and included transfontanelle ultrasonography, CT, and MRI of the brain. Infants were evaluated by a pediatric ophthalmologist at birth and every 3 months with comprehensive eye evaluations. Hearing evaluations were performed (brainstem auditory evoked response).

Based primarily on clinical assessments conducted at birth, ZIKV-exposed infants were classified as having severe, mild or moderate, or no clinical findings. Infants with ZIKV findings classified as severe had significant CNS manifestations evident at birth. The classic severe ZIKV infection phenotype may include severe microcephaly, overlapping cranial sutures, partially collapsed skull, prominent occipital bone, redundant scalp skin, severe neurological impairment, arthrogryposis, and ocular manifestations. Neuroimaging findings previously described in patients with severe ZIKV infection include intracranial calcifications, ventriculomegaly and extra-axial fluid, abnormal gyral pattern, decreased cerebral parenchymal volume, cortical atrophy and malformations, cerebellar or cerebellar vermis hypoplasia, delayed myelination, and hypoplasia or hypogenesis of the corpus callosum.^4^ Infants with ZIKV exposure classified as having mild or moderate clinical findings had neurologic symptoms at birth but were not severely affected as described, and infants with no clinical findings had a normal evaluation and no neurologic symptoms at birth.

Fetal brain disruption sequence (FBDS) describes the classic phenotypic pattern of fetal skull collapse displayed by severe microcephaly, overlapping cranial sutures, scalp skin folds (rugae), and a prominent occipital bone.^5^ Microcephaly was defined as head circumference *Z* score of less than −2 SDs for gestational age and sex at the time of birth. Severe microcephaly was defined as a head circumference *Z* score of less than −3 SDs for gestational age and sex at the time of birth. Intergrowth-21st online software, which adjusts for gestational age and sex, was used to calculate head circumference *Z* scores. Abnormal neurologic evaluation included findings such as hypertonia, hypotonia, hyperreflexia, hyporeflexia, spasticity, and seizures.

### Imaging Studies

Screening transfontanelle ultrasonography was routinely performed on ZIKV-exposed infants after birth using LOGIQ P5 (GE Medical Systems) with an 8-MHz microconvex transducer by radiologists at IFF. If abnormalities were detected or if infants were unable to have ultrasonography performed owing to small fontanelle size, infants had further CNS imaging performed (ie, CT or MRI). Infants with abnormal findings on neurologic evaluation were referred for CT and/or MRI. All ZIKV-exposed infants with available brain CT and/or MRI imaging and clinical outcome data available were included in this study. Computed tomographic brain images were obtained without sedation using the BrightSpeed Elite 16-channel (General Electric) with 120 kV and 180 mAs in a multislice helical protocol and reconstruction in axial, sagittal, and coronal planes and 3-dimensional images. Magnetic resonance imaging brain images were performed without sedation with Siemens Aera 1.5 Tesla. Most of the MRIs included T2-weighted, T1-weighted, T2*, and diffusion imaging.

### Imaging Studies Evaluation

Neuroradiographic findings of postnatal brain CT and MRI images were analyzed at the University of California, Los Angeles by 2 expert pediatric neuroradiologists, with the final interpretation determined by consensus. Two of us (N.S., a pediatric neuroradiologist with >30 years of experience, and S.K., a neuroradiologist with >5 years of postfellowship experience) independently analyzed the CT and MRI images and were blinded to clinical history; consensus was reached by discussion in cases of disagreement.

Brain CT and MRI scans were reviewed with particular focus on structural abnormalities, as opposed to nonstructural abnormalities such as hemorrhages. Structural abnormalities reviewed included reduced brain volume, brainstem hypoplasia, cerebellar hypoplasia, malformations of cortical development, brain calcification, corpus callosum abnormalities, ventriculomegaly, enlarged extra-axial cerebrospinal fluid space, enlarged cisterna magna, delayed myelination, and symmetry of abnormalities. Ventriculomegaly was classified as mild to moderate or moderate to severe based on subjective interpretation by the reviewing neuroradiologists. The location of brain calcifications was defined as cortico-subcortical white matter junction, basal ganglia, thalamus, periventricular, brainstem, or cerebellum. Infants with these neuroimaging findings or other significant findings were classified as having abnormal imaging. For infants with abnormal neuroimaging findings, the findings were evaluated to determine whether they were symmetric or asymmetric.

### Statistical Analysis

Fisher exact tests were performed to examine the association between the categorical variables and neuroimaging findings. To examine the risk of each neuroimaging abnormality and the trimester of ZIKV infection, odds ratios (ORs) and their 95% confidence intervals were reported from univariable logistic regression models in which abnormalities (yes or no) were modeled as a binary outcome and the trimester of ZIKV infection (first trimester [yes or no]) was the predictor. Sensitivity analyses were performed to study differential differences between infants’ clinical outcomes for those with ZIKV-positive PCR (yes or no). An interaction term for infants with ZIKV-positive PCR (yes or no) and abnormal imaging (yes or no) when modeling infants’ clinical outcomes (abnormal vs normal) was tested to investigate the differential difference in association between infants’ outcomes and neuroimaging abnormalities. To address the issue of sparse cell counts, the Firth penalized likelihood method was used to estimate logistic regression coefficients and *P* values. The models estimating the ORs of first trimester ZIKV infection (yes or no) were done controlling for infant ZIKV-positive PCR (yes or no). All tests were 2-sided. *P* values were not adjusted for multiple comparison, and *P* < .05 was considered statistically significant. Statistical analyses were conducted using SAS statistical software version 9.4 (SAS Institute Inc).

## Results

### Infant Cohort Characteristics

Neonatal neuroimaging (CT and/or MRI) and clinical outcome data were reviewed for 110 infants with antenatal ZIKV exposure; 81 infants (74%) had CT, 45 (41%) had MRI, and 16 (15%) had both CT and MRI. The mean (SD) gestational age for infants at birth was 38.4 (2.1) weeks (full-term). Of these infants, 11% were born preterm, and 32% were small for gestational age at birth (Table 1). There were 60 mothers with ZIKV-positive PCR testing during pregnancy, 9 infants with postnatal ZIKV-positive PCR testing, and another 12 mother-infant pairs in which both mother and infant had ZIKV-positive PCR testing; overall, 81 of 110 infants (74%) had laboratory-confirmed ZIKV exposure in pregnancy.

**Table 1.** Cohort Characteristics

Characteristic	No. (%)
No. of participants	110
Gestational age, mean (SD), wk	38.4 (2.1)
Birth measurements, mean (SD)	
Head circumference, cm	31.4 (3.7)
Weight, kg	2.9 (0.6)
Length, cm	47.5 (4.0)
Preterm	12 (11)
Small for gestational age^a^	35 (32)
History of neonatal intensive care unit	38 (35)
Cesarean delivery	64 (57)
Clinical findings	
Severe	68 (62)
Mild or moderate	6 (5)
No clinical findings	36 (33)
Fetal brain destruction sequence	50 (45)
Microcephaly	54 (49)
Congenital contractures	17 (15)
Neurologic symptoms at birth	71 (65)
Abnormal examination findings	
Eye	44 (40)
Hearing	14 (13)
Prior transfontanelle ultrasonography^b^	100 (91)
Postnatal brain computed tomography	81 (74)
Postnatal brain magnetic resonance imaging	45 (41)
Both postnatal brain computed tomography and magnetic resonance imaging	16 (15)
Age at computed tomography, median (IQR), d	14 (3-124)
Age at magnetic resonance imaging, median (IQR), d	31 (19-83)
Mothers with only PCR positive for Zika virus	60 (55)
Infants with only PCR positive for Zika virus	9 (8)
Mothers and infants both with PCR positive for Zika virus	12 (11)

Abbreviations: IQR, interquartile range; PCR, polymerase chain reaction.

^a^ Small for gestational age was defined as having a weight *Z* score less than −1.28 SDs for gestational age and sex at the time of birth.

^b^ Transfontanelle ultrasonography was not performed in 10 infants (9%). Two had normal computed tomography and/or magnetic resonance imaging findings and 8 had abnormal computed tomography and/or magnetic resonance imaging findings.

Among these 110 ZIKV-exposed infants, 68 (62%) were classified in the severe group, 6 (5%) were in the mild or moderate category, and 36 (33%) had no clinical findings. Of all exposed infants, 50 (45%) had findings of FBDS, 54 (49%) had microcephaly, and 17 (15%) had congenital contractures. In all, 71 infants (65%) had findings of neurologic symptoms at birth, 44 (40%) had abnormal ophthalmologic evaluations, and 14 (13%) had abnormal hearing evaluations (Table 1).

### Neuroimaging Findings and Infant Clinical Outcomes

**Figure 1.**
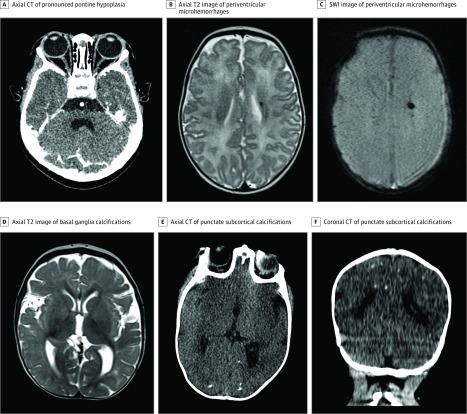
Abnormal Neuroimaging Findings in Infants Who Were Asymptomatic, Had Mild to Moderate Zika Virus Infection at Birth, or Had Normal Neurologic Evaluation Findings at Birth A, Axial contrast-enhanced computed tomography (CT) image through the brainstem demonstrates pronounced pontine hypoplasia. B, Axial T2 image demonstrates punctate susceptibility artifact along the margin of the left lateral ventricle consistent with periventricular microhemorrhages. C, Susceptibility-weighted image (SWI) demonstrates punctate susceptibility artifact along the margin of the left lateral ventricle consistent with periventricular microhemorrhages. D, Axial T2 image demonstrates punctate susceptibility artifact in the bilateral basal ganglia consistent with basal ganglia calcifications. E, Axial CT without contrast demonstrates punctate subcortical calcifications in the occipital lobes. F, Coronal CT without contrast demonstrates multiple punctate subcortical calcifications as well as a punctate periventricular calcification along the superior margin of the right lateral ventricle.

Among 110 ZIKV-exposed infants, 71 (65%) had abnormal brain imaging (MRI and/or CT) findings during the early neonatal period. Almost all of the infants with abnormal neuroimaging (96%) were classified as severely affected at birth. However, 4 of the 39 ZIKV-exposed infants with normal neurologic examinations at birth (10%) had abnormal neuroimaging findings (Figure 1; eTable 1 in the Supplement).

**Figure 2.**
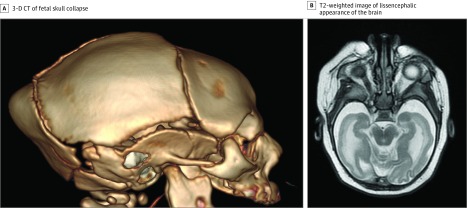
Neuroimaging Findings of an Infant With Severe Zika Virus A, Three-dimensional (3-D) computed tomography (CT) reconstruction demonstrates classic phenotypic pattern of fetal skull collapse with overlapping cranial sutures and prominent occipital protrusion. B, T2-weighted imaging demonstrates simplified gyral pattern with a lissencephalic appearance of the brain.

Abnormal imaging findings were more common among infants with ZIKV exposure with clinical findings classically associated with severe ZIKV than among those without those specific clinical features. For FBDS, 100% of infants with FBDS had abnormal imaging findings vs 35% of those with no FBDS. Results were similar for microcephaly (100% vs 30%), congenital contractures (100% vs 58%), abnormal ophthalmologic examination (95% vs 44%), abnormal hearing examination findings (100% vs 58%), and neurologic symptoms at birth (94% vs 10%) (Figure 2). For the 68 ZIKV-exposed infants with severe clinical findings and abnormal imaging findings, the most common abnormalities on neuroimaging were brain calcifications (99%), especially at the cortico-subcortical white matter junction; cortex malformations (95%), including simplified gyral patterns, pachygyria, and polymicrogyria; ventriculomegaly (93%); reduced brain volumes (87%); brainstem hypoplasia (59%); cerebellar hypoplasia (53%); and corpus callosum abnormalities (24%). When severely affected infants were subcategorized by specific clinical findings classically seen with severe ZIKV, such as FBDS, microcephaly, and congenital contractures, abnormal brain imaging findings were seen among all infants (100%) (Table 2).

**Table 2.** Clinical Outcomes and Neuroimaging Findings for Infants With Zika Virus Exposure

Infant Clinical Outcomes	Normal Neuroimaging Findings, No. (%)^a^	Abnormal Infant Neuroimaging Findings, No. (%)^a^								*P* Value^b^
		Any	Reduced Brain Volume	Brainstem Hypoplasia	Cerebellar Hypoplasia	Malformation of Cortex	Brain Calcifications	Corpus Callosum Abnormality	Ventriculomegaly	
Zika virus infection clinical classification^c^										
Severe	0	68 (100)	59 (87)	40 (59)	36 (53)	63 (95)	66 (99)	16 (24)	62 (93)	<.001
Mild or moderate	5 (83)	1 (17)	0	0	0	0	0	0	0	
No clinical findings	34 (94)	2 (6)	0	1 (3)	0	0	0	0	0	
Fetal brain disruption sequence										
Yes	0	50 (100)	48 (96)	32 (64)	28 (56)	49 (100)	48 (98)	13 (26)	47 (96)	<.001
No	39 (65)	21 (35)	11 (18)	9 (15)	8 (13)	14 (25)	18 (31)	3 (5)	15 (25)	
Microcephaly										
Yes	0	54 (100)	51 (94)	33 (61)	31 (57)	51 (96)	52 (98)	14 (26)	50 (94)	<.001
No	39 (70)	17 (30)	8 (14)	8 (14)	5 (9)	12 (23)	14 (25)	2 (4)	12 (21)	
Congenital contractures										
Yes	0	17 (100)	14 (82)	12 (71)	12 (71)	16 (100)	17 (100)	3 (18)	17 (100)	<.001
No	39 (42)	54 (58)	45 (48)	29 (31)	24 (26)	47 (52)	49 (54)	13 (14)	45 (49)	
Fundus eye examination findings										
Abnormal	2 (5)	42 (95)	36 (82)	29 (66)	26 (59)	40 (95)	41 (93)	14 (32)	41 (95)	<.001
Normal	37 (56)	29 (44)	23 (35)	12 (18)	10 (15)	23 (36)	25 (39)	2 (3)	21 (32)	
Hearing examination findings										
Abnormal	0	14 (100)	11 (79)	11 (79)	12 (86)	14 (100)	14 (100)	5 (36)	13 (100)	.002
Normal	38 (42)	52 (58)	43 (48)	27 (30)	20 (22)	47 (53)	47 (53)	9 (10)	45 (50)	
Abnormal neurologic examination findings										
Yes	4 (6)	67 (94)	59 (83)	40 (56)	36 (51)	61 (90)	64 (91)	16 (23)	62 (89)	<.001
No	35 (90)	4 (10)	0	1 (3)	0	2 (5)	2 (5)	0	0	

^a^ No significant differences for the findings were noted when the analyses were repeated to investigate differences between infant clinical outcomes and neuroimaging abnormalities for infants with and without Zika virus–positive polymerase chain reaction testing confirmed after birth.

^b^ *P* values calculated for normal neuroimaging vs any abnormal neuroimaging for infant clinical outcomes performing Fisher exact test.

^c^ Severe indicates severely affected infant with congenital Zika virus; mild or moderate indicates mild or moderate clinical findings for infants with Zika virus exposure.

Conversely, among 71 infants with abnormal neurologic examination findings at birth, 94% had abnormalities on neuroimaging, including brain calcifications (91%), cortex malformations (90%), ventriculomegaly (89%), reduced brain volumes (83%), brainstem hypoplasia (56%), cerebellar hypoplasia (51%), and corpus callosum abnormalities (23%). Among 44 infants with abnormal ophthalmologic examination findings, 95% had abnormal neuroimaging findings, and all 14 infants with abnormal hearing examination findings had abnormal neuroimaging findings (Table 2). In addition, 17 of the 56 infants with ZIKV without microcephaly at birth (30%) had significant abnormal findings on neuroimaging, which included many with brain calcifications, cortex malformations, and ventriculomegaly. Eight of these infants also had brainstem hypoplasia, and 2 had corpus callosum abnormalities.

Four ZIKV-exposed infants among 39 with normal neurologic examination findings at birth (10%) had abnormal neuroimaging findings. Two had cortical malformations and calcifications; another had isolated brainstem hypoplasia. The fourth infant had periventricular microhemorrhages and delayed myelination. In contrast, 3 of 42 infants (7%) with no or only mild-to-moderate clinical findings at birth (including eye abnormalities and/or neurologic symptoms) were found to have abnormal neuroimaging findings (Figure 1; eTables 1-3 in the Supplement). Other notable findings included 4 severely affected infants with Dandy-Walker malformation. There was also 1 infant with a left-sided middle cerebral artery infarct and another with premature metopic suture fusion with otherwise normal imaging findings.

### Neuroimaging Findings and Trimester at ZIKV Infection

The rates of neuroimaging abnormalities significantly differed by trimester at ZIKV infection. Abnormalities seen on brain CT and/or MRI were most common when ZIKV exposure occurred in the first trimester of pregnancy (63%) compared with the second (13%) and third (1%) trimesters. The odds of abnormal neuroimaging findings were nearly 8 times greater for infants with first-trimester ZIKV exposure compared with later trimesters combined (OR, 7.9; 95% CI, 3.0-20.4; *P* < .001).

More specifically, brainstem or cerebellar hypoplasia or cortex malformations (63%-67%) were more common when ZIKV occurred in the first trimester compared with the second (11%-15%) or third trimesters (0%-2%). The odds of these neuroimaging findings were greater for infants with first trimester ZIKV exposure compared with later trimesters combined (OR for brainstem hypoplasia, 3.0; 95% CI, 1.3-6.7; OR for cerebellar hypoplasia, 3.3; 95% CI, 1.4-7.6; and OR for cortex malformations, 6.6; 95% CI, 2.7-16.1; *P* < .001 for all). Similar significant findings were seen with brain calcifications, corpus callosum abnormalities, ventriculomegaly, enlarged extra-axial cerebrospinal fluid spaces, enlarged cisterna magna, and delayed myelination when analyzed by trimester at ZIKV infection. Findings of brain calcifications (OR, 6.4; 95% CI, 2.6-15.6), corpus callosum abnormalities (OR, 6.4; 95% CI, 1.7-24.2), ventriculomegaly (OR, 4.9; 95% CI, 2.1-11.4), enlarged extra-axial cerebrospinal fluid spaces (OR, 3.0; 95% CI, 1.3-7.0), enlarged cisterna magna (OR, 4.2; 95% CI, 1.9-9.5), and delayed myelination (OR, 27.0; 95% CI, 4.2-175.5) were greater when maternal ZIKV infection occurred in the first trimester, depending on the type of abnormality on neuroimaging. Symmetry of lesions did not differ by trimester at infection (Table 3).

**Table 3.** Neuroimaging Findings and Trimester of Maternal Zika Virus Infection in Pregnancy^a^

Infant Neuroimaging Findings	Trimester, No. (%)			Odds Ratio (95% CI)
	First (n = 52)	Second (n = 37)	Third (n = 5)	
Any neuroimaging abnormality	45 (63)	9 (13)	1 (1)	7.9 (3.0-20.4)
Brainstem hypoplasia	26 (63)	6 (15)	0	3.0 (1.3-6.7)
Cerebellar hypoplasia	24 (67)	4 (11)	0	3.3 (1.4-7.6)
Corpus callosum abnormality	13 (81)	1 (6)	0	6.4 (1.7-24.2)
Any cortex malformation	40 (63)	7 (11)	1 (2)	6.6 (2.7-16.1)
Simplified gyral pattern	39 (63)	7 (11)	1 (2)	4.6 (2.0-10.4)
Pachygyria	33 (62)	7 (13)	0	3.5 (1.6-7.7)
Polymicrogyria	8 (73)	1 (9)	1 (9)	6.4 (1.5-27.5)
Any brain calcifications	42 (64)	7 (11)	1 (2)	6.4 (2.6-15.6)
Cortico-subcortical white matter junction	39 (65)	7 (12)	1 (2)	5.6 (2.4-12.9)
Basal ganglia	30 (67)	6 (13)	0	4.4 (1.9-10.1)
Thalamus	19 (61)	4 (13)	0	2.5 (1.0-5.8)
Periventricular	24 (63)	4 (11)	1 (3)	2.9 (1.3-6.7)
Brainstem	10 (71)	2 (14)	0	3.3 (1.0-11.3)
Cerebellum	1 (100)	0	0	NA
Any ventriculomegaly	39 (63)	7 (11)	1 (2)	4.9 (2.1-11.4)
Mild to moderate	19 (61)	3 (10)	1 (3)	2.3 (1.0-5.5)
Moderate to severe	20 (65)	4 (13)	0	2.8 (1.2-6.5)
Enlarged extra-axial cerebrospinal fluid spaces	23 (66)	3 (9)	1 (3)	3.0 (1.3-7.0)
Enlarged cisterna magna	31 (67)	5 (11)	0	4.2 (1.9-9.5)
Delayed myelination	8 (80)	2 (20)	0	27.0 (4.2-175.5)
Symmetry of lesions				
Symmetric	44 (66)	9 (13)	1 (1)	5.7 (0.6-58.3)
Not symmetric	1 (25)	0	0	

Abbreviation: NA, not applicable.

^a^ Odds ratios were calculated using a univariable logistic regression model in which infant neuroimaging finding is the response variable and first trimester vs second and third trimesters combined (reference group) is the independent covariate. The percentages are column percentage and the denominator is the number of infants with Zika virus infection at each trimester. The trimester of infection is unknown for 16 infants. No significant differences in the findings were noted between groups when the analyses were repeated to investigate differences between neuroimaging abnormalities and trimester of maternal Zika virus infection for infants with and without Zika virus–positive polymerase chain reaction testing confirmed after birth. The direction of odds ratios and the statistical significance remained the same.

## Discussion

While our study was more heavily weighted toward the inclusion of severely affected infants with ZIKV, it is also distinguished by the inclusion of a number of ZIKV-exposed infants with either no or mild-to-moderate clinical findings. We were also able to analyze potential associations between gestational age at ZIKV infection and infant clinical data with respect to neuroimaging findings. Abnormalities on neuroimaging were commonly seen, particularly among severely affected infants with first-trimester ZIKV in utero exposure. Abnormal neuroimaging findings were uncommon among ZIKV-exposed infants with absent or mild or moderate clinical findings, including those with normal neurologic examination findings at birth.

In the 68 severely affected infants with ZIKV exposure with abnormal imaging findings, nearly all had brain calcifications, cortical malformations, ventriculomegaly, and reduced brain volumes; many also demonstrated brainstem or cerebellar hypoplasia and corpus callosum abnormalities. The frequency of neuroimaging abnormalities seen in our study paralleled those seen in other studies of severely affected infants where high rates of calcifications (93%-100%), cortical malformations (69%-100%), ventriculomegaly (66%-96%), cerebellar abnormalities (65%-82%), and brainstem hypoplasia (21%-70%) were observed.^4,5,6,7,8,9,10,11^ Rates of specific abnormalities on neuroimaging appeared similar when analyzed by particular clinical features of severe ZIKV such as microcephaly, FBDS, congenital contractures, and ophthalmologic and hearing abnormalities. As prior studies^5,6,12^ have noted, calcifications at the cortico-subcortical white matter junction, which are thought to be more characteristic of ZIKV infection compared with other congenital infections, were commonly seen in this cohort of infants with ZIKV exposure. We found these types of brain calcifications among 60 of 66 infants with calcifications (91%). These were the most common type of calcification seen in the infants. While calcifications of the basal ganglia seen in some of the infants in this study are often considered rarer findings in congenital infections and have been reported in other noninfectious conditions, such as Aicardi-Goutieres syndrome,^13^ these types of calcifications have been previously identified in ZIKV infants.^5^ Soft-tissue calcifications have been shown to be mediated by cells undergoing osteogenic fates, similar to physiological bone formation.^14^ Brain malformations from ZIKV are thought to result from apoptosis of neuroprogenitor cells followed by microglial reaction, and calcifications may represent calcium deposition following this process.^15,16,17^ While the etiology of ZIKV-associated calcification is not yet known, it has been suggested that these calcium depositions at the cortico-subcortical junction could be due to vasculopathy resulting from ZIKV infection.^5^ Future studies to address the mechanisms of congenital ZIKV calcifications and birth abnormalities are warranted.

One Brazilian neuroimaging study^6^ of infants with severe congenital ZIKV infection observed that the most devastating neuroimaging findings were seen in ZIKV-infected infants with microcephaly, and findings such as brainstem hypoplasia were only seen in their more severe ZIKV cases. However, 1 infant with ZIKV exposure in our cohort had isolated brainstem hypoplasia but no other abnormalities on neuroimaging. The infant had normal neurologic examination findings at birth and no other clinical findings apart from abnormal findings on a hearing evaluation at a later follow-up visit. However, at 1 year, this child had neurodevelopmental delay (eTable 1 and eTable 2 in the Supplement). In fact, 8 of 56 infants with ZIKV exposure without microcephaly at birth (14%) had brainstem hypoplasia and 2 (4%) had corpus callosum abnormalities. Previously, these findings were only reported by other groups in severe cases of congenital ZIKV with microcephaly. These findings seem to underscore the importance of neuroimaging (CT and/or MRI) for infants with abnormalities on transcranial focused ultrasonography or clinical evaluation even if microcephaly is not present.

Other findings of interest included 1 infant with ZIKV-positive PCR results who had hemiparesis, developmental delay, and seizures after delivery and was found to have a left middle cerebral artery infarct (parietal, occipital, temporal) (eTable 2 in the Supplement). Cerebral infarcts have been reported in other congenital infections, especially varicella,^18^ but rarely observed following ZIKV infection; 2 cases reported infants with ZIKV exposure with cerebral infarct, including an infant with a left middle cerebral artery infarct, and were possibly attributed to congenital ZIKV.^19,20^ Dandy-Walker malformation, a rare condition characterized by hypoplasia or agenesis of the cerebellar vermis, cystic dilation of the fourth ventricle, and posterior fossa enlargement, was seen in 4 of 110 ZIKV-exposed infants (4%), all of whom also had other significant neuroimaging abnormalities, including calcifications. Three of the infants were born to mothers with ZIKV-positive PCR results in pregnancy, and 2 infants themselves had ZIKV-positive PCR results after birth. Dandy-Walker malformation has been reported previously in other congenital infections, especially congenital rubella,^21^ and observed in several other ZIKV radiology reports.^5,10,22,23^

Classification as ZIKV with normal neurologic examination findings or either no or mild to moderate clinical findings at birth tended to preclude abnormal neuroimaging findings. However, some exceptions were observed. Four of 39 ZIKV-exposed infants with normal neurologic examination findings at birth (10%) had abnormal neuroimaging findings. For the 2 infants with cortical malformations and calcifications, it was unclear from the history if the infants’ neurologic examination findings at birth were truly normal because the infants had been born at an outside hospital and records were not detailed. Nevertheless, our findings highlight some limitations in relying on infant clinical birth evaluations. The infant with periventricular hemorrhages had perinatal asphyxia at birth and later developmental delay with autism noted later in life. In this setting, evaluating ZIKV’s association with these findings is complex. It also remains to be determined whether infants with ZIKV with normal neuroimaging findings at birth or only minor, nonstructural abnormalities will change over time on subsequent evaluations.

In the present study, the likelihood of abnormalities on neuroimaging also differed significantly by trimester at ZIKV infection. Abnormal brain imaging findings were most common when ZIKV occurred in the first trimester (63%) compared with the second trimester (13%) and third trimester (1%). The risk of neuroimaging abnormalities associated with ZIKV infection in the first trimester compared with infection in later trimesters combined was significantly higher (OR, 7.9; 95% CI, 3.0-20.4), and these infants consistently demonstrated nearly all types of neuroimaging abnormalities. Prior studies^3,4,24,25^ have demonstrated that congenital neurologic abnormalities are more likely to be seen following ZIKV infection in the first trimester of pregnancy. Epidemiologic studies conducted by the Centers for Disease Control and Prevention have also suggested that characteristic ZIKV neurologic or ocular abnormalities range between 5% and 10% but are as high as 8% to 15% when ZIKV infection occurs in the first trimester.^24,26,27^ As with many other congenital infections, including cytomegalovirus and rubella, early trimester in utero infection is associated with more severe clinical manifestations given viral teratogenic exposure during fetal development.^12^ Our findings are consistent with this.

### Limitations

This study has several limitations. One limitation was that our ZIKV-exposed infant population was heavily weighted toward severely affected infants, as those with abnormal findings on head ultrasonography or neurologic evaluations were referred to CT and/or MRI imaging. Thus, most infants had a clinical indication for additional imaging and may reflect potential selection biases that may limit generalization of these findings, particularly for less severely affected infants. In addition, while CT is the modality of choice for evaluation of calcifications and MRI may be better for evaluation of other types of structural brain abnormalities, most ZIKV-exposed infants were not able to have both CT and MRI given limited resources for evaluation and workup of these infants in an epidemic setting. As serial imaging was not available at the time of this study, we were unable to assess whether changes were present over time in severely affected infants with structural CNS abnormalities. We were also unable to assess whether the infants had resolving calcifications, such as those demonstrated in prior reports.^15^ This study’s cohort, composed of all children followed at a referral center for ZIKV who had CT or MRI neuroimaging performed, differs from our strictly prospective ZIKV-exposed pediatric longitudinal cohort.^3,25,28^ Therefore, frequency data from the present study may not necessarily reflect incidence data.

## Conclusions

Abnormalities on neuroimaging (CT and/or MRI) were commonly seen in ZIKV-exposed infants followed up at a maternity and children’s hospital ZIKV referral center in Rio de Janeiro. These structural CNS abnormalities occurred primarily in severely affected infants, especially when ZIKV infection occurred in the first trimester of pregnancy. Brain calcifications, especially at the cortico-subcortical white matter junction, cortex malformations, ventriculomegaly, and reduced brain volumes were most frequently seen. Neuroimaging of antenatally ZIKV-exposed infants is an important component in a comprehensive evaluation that may help identify and determine the extent of CNS involvement associated with in utero ZIKV infection.

## Notes

Supplement. **eTable 1.** Zika Infants With Normal Neurologic Evaluation at Birth and Abnormal Neuroimaging **eTable 2.** Zika Infants With No or Mild/Moderate Clinical Findings With Abnormal Neuroimaging **eTable 3.** Summary of the Types of Mild to Moderate Clinical Findings for Zika Infants
