# Supplementary material for: Association Between Neonatal Neuroimaging and Clinical Outcomes in Zika-Exposed Infants From Rio de Janeiro, Brazil
Source: JAMA Netw Open. 2019 Jul 31;2(7):e198124. doi: 10.1001/jamanetworkopen.2019.8124 (PMC6669783; doi:10.1001/jamanetworkopen.2019.8124)
Supplement: Supplement. — eTable 1. Zika Infants With Normal Neurologic Evaluation at Birth and Abnormal Neuroimaging eTable 2. Zika Infants With No or Mild/Moderate Clinical Findings With Abnormal Neuroimaging eTable 3. Summary of the Types of Mild to Moderate Clinical Findings for Zika Infants [file jamanetwopen-2-e198124-s001.pdf]

Supplementary Online Content

Pool K-L, Adachi K, Karnezis S, et al. Association between neonatal neuroimaging and clinical outcomes in Zika-exposed infants from Rio de Janeiro, Brazil. *JAMA Netw Open*. 2019;2(7):e198124. doi:10.1001/jamanetworkopen.2019.8124

eTable 1. Zika Infants With Normal Neurologic Evaluation at Birth and Abnormal Neuroimaging
eTable 2. Zika Infants With No or Mild/Moderate Clinical Findings With Abnormal Neuroimaging
eTable 3. Summary of the Types of Mild to Moderate Clinical Findings for Zika Infants

This supplementary material has been provided by the authors to give readers additional information about their work.

© 2019 Pool K-L et al. *JAMA Network Open.*

eTable1: : Zika Infants with Normal Neurologic Evaluation at Birth and Abnormal Neuroimaging

Infant	Summary of Clinical and Neuroimaging Findings
**Infant 1***	Considered to have no abnormal clinical findings consistent with a Zika at birth. However, preterm at 35 weeks gestational age and presented with asphyxia at birth with respiratory distress and possible neonatal sepsis. Later noted to have speech delay, possible autism during early childhood.<br>Supposedly normal neurologic exam at birth, eye exam, hearing evaluation normal.<br>Periventricular microhemorrhages and delayed myelination were the only abnormalities noted on neuroimaging.
**Infant 2***	Considered to have no abnormal clinical findings consistent with Zika at birth. Later noted to have abnormal hearing exam and also found to have neurodevelopmental delay at 12 months of age.<br>Only brainstem hypoplasia noted on neuroimaging.
**Infant 4**	Found to have a normal neurologic exam at birth, normal eye, and hearing evaluation. However, later noted to have abnormal neurologic findings during infancy and our team suspects that the infant possibly may have had an abnormal neurologic exam at birth when evaluated at the outside hospital.<br>Malformation of cortical development including simple gyral patterns and calcifications at the subcortical white matter junction were noted on neuroimaging.
**Infant 5**	Found to have a normal neurologic exam at birth, normal eye, and hearing evaluation at outside hospital. When evaluated by our team after birth, found to have microcephaly, occipital prominence at 5 months of age and later developed seizures and abnormal neurologic exam. Our team suspects that clinical evaluation at birth may have been abnormal at outside hospital.<br>Malformation of cortical development (simple gyral pattern, pachygyria, polymicrogyria) as well as calcifications (cortico-subcortical white matter junction, basal ganglia, parenchymal) were noted on neuroimaging.

eTable 2: Zika Infants with No or Mild/Moderate Clinical Findings with Abnormal Neuroimaging

Infant	Summary of Clinical and Neuroimaging Findings
**Infant 1***	Considered to have no abnormal clinical findings consistent with Zika at birth. However, preterm at 35 weeks gestational age and presented with asphyxia at birth with respiratory distress and possible neonatal sepsis. Later noted to have speech delay, possible autism during early childhood.<br>Supposedly normal neurologic exam at birth, eye exam, hearing evaluation normal.<br>Possible periventricular microhemorrhages and delayed myelination was the only abnormality noted on neuroimaging.
**Infant 2***	Considered to have no abnormal clinical findings consistent with Zika at birth. Later noted to have abnormal hearing exam and also found to have neurodevelopmental delay at 12 months of age.<br>Only brainstem hypoplasia noted on neuroimaging.
**Infant 3**	Considered to have abnormal clinical findings at birth with abnormal neurologic exam with brain ischemia, hemiparesis, neurodevelopmental delay, and seizures at 15 hours of life. However, these findings are not classically associated with severe congenital Zika infection described in the literature.<br>Left-sided middle cerebral artery (MCA) infarct noted on neuroimaging.

**Note: Infants 1&2 are in both asymptomatic and normal neurologic exam at birth groups.*

© 2019 Pool K-L et al. *JAMA Network Open.*

eTable 3: Summary of the Types of Mild to Moderate Clinical Findings for Zika Infants

**Mild to Moderate Clinical Findings**
SGA, abnormal fundoscopic eye evaluation, abnormal hearing evaluation, abnormal neurologic evaluation including irritability, hyper/hypotonia, hyper/hyporeflexia, spasticity, and seizures
***Please note there was also 1 infant described in appêndix Table 1 with L MCA infarct who also had hemiparesis, seizures, and neurodevelopmental delay*

© 2019 Pool K-L et al. *JAMA Network Open.*
